# The Significance of Low Titre Antigliadin Antibodies in the Diagnosis of Gluten Ataxia

**DOI:** 10.3390/nu10101444

**Published:** 2018-10-05

**Authors:** Marios Hadjivassiliou, Richard A Grünewald, David S Sanders, Panagiotis Zis, Iain Croall, Priya D Shanmugarajah, Ptolemaios G Sarrigiannis, Nick Trott, Graeme Wild, Nigel Hoggard

**Affiliations:** 1 Academic Departments of Neurosciences and Neuroradiology, Sheffield Teaching Hospitals NHS Trust, Sheffield S10 2JF, UK; r.a.grunewald@sheffield.ac.uk (R.A.G.); takiszis@gmail.com (P.Z.); i.croall@sheffield.ac.uk (I.C.); p.d.shanmugarajah@sheffield.ac.uk (P.D.S.); p.sarrigiannis@sheffield.ac.uk (P.G.S.); n.hoggard@sheffield.ac.uk (N.H.); 2 Departments of Gastroenterology, Sheffield Teaching Hospitals NHS Trust, Sheffield S10 2JF, UK; David.Sanders@sth.nhs.uk (D.S.S.); 3 Departments of Dietetics, Sheffield Teaching Hospitals NHS Trust, Sheffield S10 2JF, UK; Nick.Trott@sth.nhs.uk (N.T.); 4 Departments of Immunology, Sheffield Teaching Hospitals NHS Trust, Sheffield S10 2JF, UK; Graeme.Wild@sth.nhs.uk (G.W)

**Keywords:** Gluten ataxia, antigliadin antibodies, coeliac disease, MR spectroscopy, gluten sensitive enteropathy, antigliadin antibody titre

## Abstract

Background: Patients with gluten ataxia (GA) without enteropathy have lower levels of antigliadin antibodies (AGA) compared to patients with coeliac disease (CD). Magnetic Resonance Spectroscopy (NAA/Cr area ratio) of the cerebellum improves in patients with GA following a strict gluten-free diet (GFD). This is associated with clinical improvement. We present our experience of the effect of a GFD in patients with ataxia and low levels of AGA antibodies measured by a commercial assay. Methods: Consecutive patients with ataxia and serum AGA levels below the positive cut-off for CD but above a re-defined cut-off in the context of GA underwent MR spectroscopy at baseline and after a GFD. Results: Twenty-one consecutive patients with GA were included. Ten were on a strict GFD with elimination of AGA, 5 were on a GFD but continued to have AGA, and 6 patients did not go on a GFD. The NAA/Cr area ratio from the cerebellar vermis increased in all patients on a strict GFD, increased in only 1 out of 5 (20%) patients on a GFD with persisting circulating AGA, and decreased in all patients not on a GFD. Conclusion: Patients with ataxia and low titres of AGA benefit from a strict GFD. The results suggest an urgent need to redefine the serological cut-off for circulating AGA in diagnosing GA.

## 1. Introduction

Gluten ataxia (GA) is defined as otherwise idiopathic sporadic ataxia with serological evidence of gluten sensitivity in the absence of an alternative cause [1]. The presence or absence of an enteropathy is not a prerequisite for its diagnosis [2]. Indeed, up to 50% of patients with GA do not have an enteropathy, yet they still benefit from a gluten-free diet (GFD) [2]. For this reason, IgG and IgA native antigliadin antibodies (AGA) are currently the most sensitive marker for GA when compared to endomysium (EMA) and transglutaminase 2 antibodies (TG2), both of which are specific for the presence of enteropathy (Coeliac Disease-CD) [2]. Despite this, the majority of immunology laboratories in the UK and other countries have abandoned the use of native AGA assays in the diagnosis of CD because of poor specificity. Estimation of specificity, however, is based on the presence of a gold standard, in this case the presence of enteropathy (CD). It is now widely accepted that sensitivity to gluten can be present without enteropathy [3]. The only current serological biomarker helpful in diagnosing gluten sensitivity without enteropathy is AGA [4].

Patients with CD often have high titres of circulating AGA, whereas patients with GA and no enteropathy tend to have low titres. The serological cut-off for significant titre in commercially available AGA assays is calculated to maximize diagnostic specificity using data from patients with CD. This would not necessarily be applicable to those patients with gluten sensitivity who do not have enteropathy and those patients with extraintestinal manifestations.

Having previously demonstrated the beneficial effect of a GFD in patients with GA using MR spectroscopy of the cerebellum, in this report we present our experience of the effect of a GFD in patients with ataxia and AGA levels that are below what is considered positive, as defined by the manufacturer, but above a newly defined cut-off AGA level based on our extensive experience in managing patients with GA and the re-evaluation of over 500 patients with GA.

## 2. Methods

This report is based on prospective observational case series of patients regularly attending the gluten sensitivity/neurology clinic run by one of the authors (M.H.). The South Yorkshire Research Ethics Committee has confirmed that no ethical approval is indicated given that a gluten-free diet is a recognized treatment for suspected patients with GA and that all investigations/interventions were clinically indicated and did not form part of a research study.

### 2.1. AGA Serological Testing

In January 2015, the immunology lab at Sheffield Teaching Hospitals NHS Trust changed the ELISA AGA (IgG and IgA) assay to Phadia 2500 [5]. The decision was based on the benefits of an automated high throughput process. After consultation with the clinicians using the AGA assay, a decision was made to provide numerical values for the AGA results instead of just positive/negative results.

The manufacturers provided the following information regarding positivity (for both IgA and IgG) of their assay: 0–7 U/mL negative, 7–10 U/mL borderline, >10 U/mL positive.

### 2.2. Patient Selection and Follow-up

The Sheffield Ataxia Centre cares for over 1800 patients with progressive ataxia, including over 500 patients with GA. All patients with ataxia undergo extensive investigations to try and identify a cause [6]. Such investigations include serological testing for AGA, EMA and TG2. Our previous report on the effect of a GFD on MR spectroscopy in 117 patients with GA was based on those patients with AGA serological positivity (with or without serological positivity for TG2 and EMA antibodies and/or enteropathy) using previous commercial AGA assays by our immunology lab, or those patients who had a value of over 7 U/mL using the new assay [7]. Fifty percent of our cohort of patients with GA have an enteropathy (CD). As expected in patients with enteropathy, EMA and TTG antibodies are also positive. All patients diagnosed with GA at our centre are routinely advised to adopt a strict GFD and are referred to an experienced dietitian for GFD advice. Strict adherence to a GFD is assumed when there is elimination of all gluten related antibodies. If patients have persistently positive antibodies, they are reviewed by an experienced dietitian (NT) for further advice. Patients who still have persistently positive AGA are assumed not to be strict with a GFD.

In the current report we have included only those consecutive patients with serological results for IgG and/or IgA AGA over 3 U/mL but less than 7 U/mL. The lower cut-off value of 3 U/mL was derived based on our experience in the diagnosis and management of over 500 patients with GA who regularly attend the Sheffield Ataxia Centre and either did not adopt a GFD or were on a partial (non-strict) GFD. All of these patients were repeatedly tested using the new assay, irrespective of their GFD status. The new serological cut-off was also based on AGA estimation in those GA patients already on a strict GFD who had persistently absent circulating AGA, using previous assays and with evidence of improvement of their ataxia clinically and on MR spectroscopy. All of these patients with GA who remained neurologically stable had values below 3 U/mL on the new assay. Patients on partial or no GFD had levels above 3 U/mL but often less than 7 U/mL. We therefore used the cut-off of 3 U/mL, below which we assumed strict adherence to a GFD.

Consecutive patients included in this report attend the Sheffield Ataxia Centre on a 6 monthly basis. All patients had undergone more than one clinical MR spectroscopy scan for the purpose of diagnosis and monitoring of their ataxia since 2015, the year of the introduction of the new AGA assay. Only a third of the patients reported here underwent gastroscopy and duodenal biopsy to establish the presence of enteropathy (triad of villus atrophy, crypt hyperplasia and increased intraepithelial lymphocytes). This was based on patient choice after informing them of their serological results, including the negative serology for TG2 and EMA antibodies. None of these patients had enteropathy as predicted by the negative EMA and TG2 antibodies, but presence or not of enteropathy was not an exclusion criterion. Patients were reviewed by an experienced dietitian (NT) who provided detailed advice on a GFD with further monitoring by telephone or face-to-face consultations. The patients underwent repeat clinical evaluation and further serological testing at approximately 6 monthly intervals. Strict adherence to a GFD was indicated by serological elimination of circulating AGA (<3 U/mL). For the purpose of this report, the patients were divided into 3 groups: those with strict adherence to GFD with AGA levels of <3 U/mL, those with partial adherence to a GFD as evident from the presence of AGA (above 3 U/mL but less that 7 U/mL), and a third group consisting of patients that declined GFD (AGA level above 3 U/mL and less than 7 U/mL).

We also reviewed the AGA results in patients with classical CD presenting to gastroenterology clinics and patients with idiopathic sporadic ataxia. Data on the prevalence of AGA (range between 3–7 U/mL) was also available from healthy volunteers as part of another ongoing study.

### 2.3. MR Spectroscopy

In addition to volumetric 3T MR imaging, all patients underwent single-voxel H^1^ MR spectroscopy of the cerebellum. This imaging protocol is in clinical use as part of the investigation of all patients with cerebellar ataxia attending the Sheffield Ataxia Centre. The brain imaging protocol for structural, volumetric, and spectroscopy studies has been previously described [7]. The main measurement is the NAA/Cr area ratio within the cerebellar vermis. N-acetyl aspartate (NAA) reflects the health of neurons and is a reliable marker of monitoring neuronal energy impairment and dysfunction. Creatine (Cr) is a stable metabolite with little variation between different pathologies. As such, it is typically used as an internal standard in MR spectroscopy from which metabolite ratios can be calculated.

A baseline MR spectroscopy scan was done on all patients and a repeat scan was done after the introduction of the GFD. In common with other immune-mediated ataxias, the cerebellar vermis is primarily involved in GA; therefore, MR spectroscopy results reported here are measurements from the cerebellar vermis.

### 2.4. Statistical Analysis

Change in mean values between the groups was compared with Student’s two-tailed *t*-test for unpaired samples. A value of *p* < 0.05 was considered significant.

## 3. Results

A total of 21 consecutive patients with GA were included at the time of writing this report. Detailed clinical characteristics of patients with GA have been described previously. The patients included in this report did not differ in any way from those patients with GA described previously [1,8].

**Figure 1:**
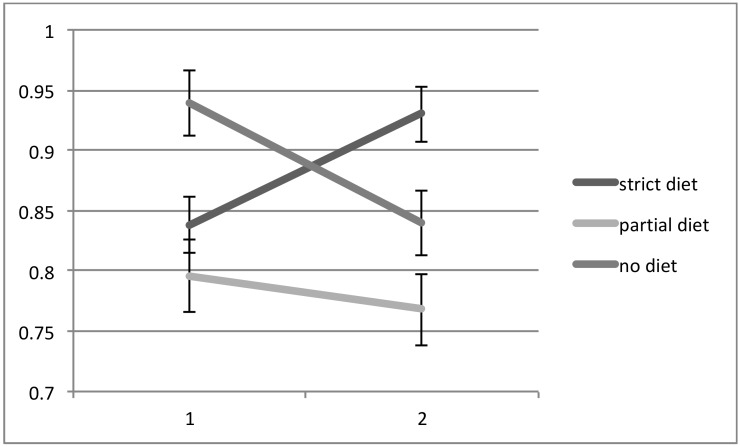
NAA/Cr area ratio in patients with gluten ataxia without enteropathy following the introduction of a gluten-free diet (GFD) in the 2 sub-groups (number of patients on gluten free diet 10, partial diet 5). The third subgroup consisted of 6 patients who declined a GFD. All patients had antigliadin antibodies (AGA) IgG and/or IgA of >3 U/mL and <7 U/mL at baseline. Both the partial diet and no diet groups still had AGA values >3 U/mL at the time of the second scan. All 10 patients on a strict GFD showed improvement of the NAA/Cr area ratio (vertical axis) of the vermis 4 of the 5 patients on partial GFD and all 6 patients not on GFD showed deterioration of the NAA/Cr ratio. A Chi squared contingency table looking at numbers improved on magnetic resonance spectroscopy on a strict diet compared with no diet was significant *p* < 0.0001.

**Figure 2:**
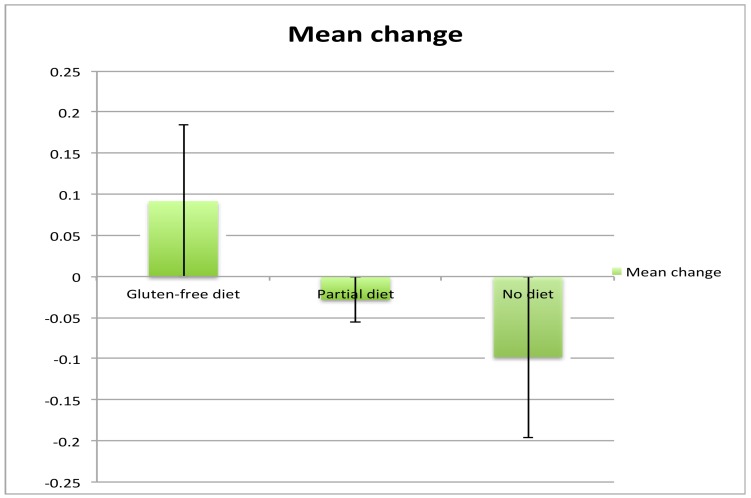
Mean change of the area ratio of NAA/Cr from baseline in the 3 groups.

All patients had two MR spectroscopy scans at baseline and the second after a mean interval of 19 months (range 5 months to 36 months). Of the 21 patients, 10 were on a strict GFD with elimination of all antibodies (IgG and/or IgA AGA <3 U/mL), 5 were on a GFD but still had serological evidence of circulating AGA, indicating ongoing exposure to gluten (IgG and/or IgA >3 U/mL), and 6 patients were not on the diet (IgG and/or IgA AGA >3 U/ml and <7 U/mL). There were no significant differences in the duration of ataxia between the 3 groups. The patients on partial GFD were significantly younger that the other 2 groups. There were, however, no significant differences in age between the strict GFD and no GFD groups. Those patients that declined a GFD also had a repeat scan for monitoring purposes. The NAA/Cr area ratio taken from the cerebellar vermis increased in all 10 patients on a strict diet, but in only 1 out of 5 (20%) patients on a partial GFD with persistent circulating AGA. In the remaining 4, the NAA/Cr area ratio decreased. In all of the 6 patients not on a diet, there was a decrease in NAA/Cr area ratio on repeat scanning. These results are illustrated in Figure 1 and Figure 2. A Chi squared contingency table looking at numbers improved on MR spectroscopy on a strict diet compared with no diet was significant *p* < 0.0001. A comparison of the change in MR spectroscopy values from baseline between the 3 groups showed the following: no diet mean change −0.098, Standard error of the Mean (SEM) 0.06, partial diet mean change −0.028, SEM 0.087 and strict diet mean change +0.092, SEM 0.06. Comparison between the strict diet and no diet groups was significant (*p* < 0.0001), as was the comparison between partial diet and no diet (*p* = 0.0028).

Using the same serological cut-off for AGA titres of >3 U/mL, the prevalence of AGA positivity amongst 68 patients with classical CD presenting to the gastroenterologists was 100%. The mean baseline value for AGA titre in this classical CD group was 46.5 U/mL. This compares to a mean AGA titre of 4.1 U/mL in the 21 patients reported here. The prevalence amongst 28 healthy controls was 7%, and the prevalence amongst 197 patients with otherwise idiopathic sporadic ataxia was 39%. This group of 197 did not include patients with ataxia who had AGA levels over 7 U/mL or those who also had enteropathy. Table 1 summarises the clinical and serological characteristics of the GA groups.

## 4. Discussion

We have previously demonstrated clinical and MR spectroscopic improvement in patients with GA after a year of strict adherence to a gluten-free diet [7,9]. The current study demonstrates that in patients with GA with low titres of AGA, NAA/Cr area ratio within the cerebellar vermis improves with strict adherence to a gluten-free diet, worsens with on-going exposure to gluten, and also largely worsens with partial adherence to a gluten-free diet, as indicated by persistently positive circulating AGA. The improvement in MR spectroscopy was accompanied by clinical improvement or stabilisation of the ataxia in the strict GFD group.

The advantage of MR spectroscopy as a monitoring tool is that it can be easily performed as part of routine MR imaging, it is reproducible in an individual on a particular scanner, and relies on objective measurements such as the NAA/Cr area ratio [7,10]. It therefore overcomes the disadvantages of the clinical scales (interrater variability, fluctuation of ataxia symptoms and signs due to fatigue, insensitive scales in disabled patients and ceiling effect). Other groups have also demonstrated good correlation between MR spectroscopy and the clinical status as assessed by ataxia rating scales [11]. This report also demonstrates the importance of strict adherence to the GFD. Amongst those patients on the diet, but not strict, only 20% improved on MR spectroscopy as opposed to 100% in those on a strict GFD. Strict diet with serological evidence of elimination of all antibodies appears to have the potential to stabilise and partially reverse immune mediated damage to the cerebellum.

This report also demonstrates that for extraintestinal manifestations of gluten sensitivity, and in particular for those patients without enteropathy, the level of circulating AGA that is still significant is lower than that seen in the context of enteropathy. This means that the serological cut-off titre for diagnosing GA requires adjustment. This has major implications for the diagnosis of GA. Our data from 197 patients with idiopathic sporadic ataxia collected since 2015 (the year of the introduction of the new assay), excluding those with positive AGA (using the manufacturer’s serum cut-off level for positivity) and those with CD, showed that 39% had AGA levels between 3 and 7 U/mL. As we have shown here, these patients also benefited from a strict GFD. Up until now, such patients would have remained undiagnosed and therefore followed a progressive course as a result of ongoing exposure to gluten. Indeed, some of these patients had been under regular follow-up in our ataxia clinic but were negative for AGA on previous assays used by our immunology lab.

We have found MR spectroscopy a reliable and useful tool in the monitoring of patients with GA and other ataxias. Improvement of NAA/Cr in patients adherent to a GFD bolsters the diagnosis of GA and, in our experience, is accompanied by clinical improvement [9]. Such improvement also acts as a motivation for patients to continue with the GFD. The commonest cause for the lack of improvement tends to be poor adherence to the GFD.

Both this report and previous publications from our group have highlighted the importance of using the correct serological markers for the diagnosis of GA. The presence of enteropathy (associated with the presence of positive TG2 and endomysium antibodies) does not influence the response to the GFD, and thus any patient with positive antigliadin antibodies and no other cause of ataxia should be offered a strict gluten-free diet, even in the absence of enteropathy.

In conclusion, using MR spectroscopy data we have demonstrated that patients with ataxia and low titre of AGA improve on a strict GFD. We are therefore proposing a new AGA titre cut-off level that should be used in the diagnosis of GA.

## Figures and Tables

**Table 1:** Summary of clinical characteristics and the change in magnetic resonance spectroscopy and antigliadin antibody titre at baseline and at the time of the second scan in the 3 groups. The differences in numbers improved and in the changes in MR spectroscopy between the strict GFD and the not on GFD groups were significant *p* < 0.0001. There were no significant clinical differences between the strict GFD and the partial or not on diet groups. The patients on partial GFD were significantly younger than the other 2 groups. GA = gluten ataxia; NAA/Cr = N Acetyl Aspartate to Creatine ratio; GFD = Gluten Free Diet; AGA = Antigliadin antibodies; SD standard deviation.

Dietary Status in GA Groups	Numb per Group	Mean Age	Mean Duration of Ataxia in Years	Mean MR Spectroscopy Change from Baseline (NAA/Cr Area Ratio)	Numb Improv-ed	Mean AGA Antibody Titre at Baseline (SD)	Mean AGA Antibody Titre at the Time of 2nd Scan (SD)
strict GFD	10	65	6.4	+0.092	10	3.6 (0.46)	1.8 (0.74)
partial GFD	5	50	6.6	−0.028	1	4.5 (1.3)	3.8 (1)
not on diet	6	77	7.3	−0.098	0	4.2 (1.3)	4.2 (1.8)
